# Causal effects of gut microbiota on diabetic neuropathy: a two-sample Mendelian randomization study

**DOI:** 10.3389/fendo.2024.1388927

**Published:** 2024-08-02

**Authors:** Ming Xu, Jinxuan Hao, Yijie Qi, Baofeng Wu, Ru Li, Xifeng Yang, Yi Zhang, Yunfeng Liu

**Affiliations:** ^1^ Department of Endocrinology, First Hospital of Shanxi Medical University, Taiyuan, Shanxi, China; ^2^ The First Clinical Medical College, Shanxi Medical University, Taiyuan, Shanxi, China; ^3^ Department of Pharmacology, School of Basic Medicine, Shanxi Medical University, Taiyuan, Shanxi, China; ^4^ Medicinal Basic Research Innovation Center of Chronic Kidney Disease, Ministry of Education, Shanxi Medical University, Taiyuan, Shanxi, China

**Keywords:** diabetic neuropathy, Mendelian randomization, GWAS - genome-wide association study, diabetic autonomic nervous system neuropathy, diabetic polyneuropathy

## Abstract

**Objective:**

Previous observational studies have suggested an association between gut microbiota and diabetic neuropathy (DN). However, confounding factors and reverse causality make the causal relationship between gut microbiota and DN uncertain. We aimed to investigate the interactive causal relationships between the abundance of gut microbiota and DN.

**Methods:**

We conducted a Mendelian randomization (MR) analysis to examine the causal relationship between gut microbiota and DN. Genomic data on gut microbiota at the genus level were obtained from the MiBioGen Consortium, including 18,340 individuals of European descent. Data on diabetic polyneuropathy (DPN) were obtained from the FinnGen Consortium, which included 1,048 cases and 374,434 controls, while data on diabetic autonomic neuropathy (DAN) were also obtained from the FinnGen Consortium, including 111 cases and 374,434 controls. Causal effects were primarily estimated using inverse variance weighted (IVW) analysis, supplemented with four validation methods, and additional sensitivity analyses to assess the pleiotropy, heterogeneity, and robustness of instrumental variables.

**Results:**

The IVW analysis indicated that *Prevotella 9* had a protective effect on DPN (OR = 0.715, 95% CI: 0.521-0.982, P = 0.038), and *Bacteroides* also showed a protective effect (OR = 0.602, 95% CI: 0.364-0.996, P = 0.048). On the other hand, *Ruminococcus 2* had a promoting effect on DPN (OR = 1.449, 95% CI: 1.008-2.083, P = 0.045). *Blautia* (OR = 0.161, 95% CI: 0.035-0.733, P = 0.018), *Clostridium innocuum group* (OR = 3.033, 95% CI: 1.379-6.672, P = 0.006), and *Howardella* (OR = 2.595, 95% CI: 1.074-6.269, P = 0.034) were causally associated with DAN in the IVW analysis, with no evidence of heterogeneity or pleiotropy. Sensitivity analyses showed no significant pleiotropy or heterogeneity.

**Conclusion:**

Our study identified a causal relationship between gut microbiota and the increased or decreased risk of diabetic neuropathy. These findings underscore the importance of adopting a comprehensive approach that combines gut microbiota modulation with other therapeutic interventions in the management of diabetic neuropathy.

## 1 Introduction

Peripheral diabetic neuropathy (PDN) is a common complication of diabetes that results in functional damage to the peripheral nervous system, primarily caused by hyperglycemia. The majority of PDN cases are classified as diabetic polyneuropathy (DPN), which can cause pain, stinging, prickling sensations, and numbness that may progress to complete loss of sensation and motor dysfunction (1). Meanwhile, a portion of DN cases are categorized as diabetic autonomic neuropathy (DAN), which is associated with poor prognosis among patients who exhibit its symptoms.

While there are multiple factors that contribute to the development of DPN, including oxidative stress, accumulation of late glycation end products, blood flow disorders, and altered growth factor expression, chronic hyperglycemia-induced oxidative stress is considered a key mediator leading to the occurrence and progression of DPN (2, 3). Currently, effective therapies for PDN are rare, and thus, it is urgent to explore new treatment methods (4). Interestingly, not all diabetic patients develop neuropathy, and the underlying mechanism remains unexplained.

Many studies suggest that changes in gut microbiota composition and metabolites may lead to the occurrence of obesity and related metabolic disorders, which can be potential therapeutic targets for such diseases (5, 6). Additionally, correlations have been reported between gut microbiota regulation and improvements in oxidative stress, inflammatory response, and insulin resistance, which could affect diabetes complications (7). Experiments have shown that colonization with normal and healthy gut microbiota can restore the homeostasis and neuronal activity of the gut nervous system in germ-free mice, whereas gut microbiota from diabetic mice have the opposite effect (8, 9). However, mechanistic studies of the microbiome are often difficult to conduct on humans due to the heterogeneity of genetic and lifestyle factors and ethical issues related to human subjects that may lead to disease-causing microbial colonization (10). In our study, we employed a two-sample Mendelian randomization (MR) analysis to investigate the causal relationship between microbiota and diabetic peripheral neuropathy (DPN) and diabetic autonomic neuropathy (DAN).

## 2 Materials and methods

### 2.1 Hypothesis and study design of MR

**Figure 1:**
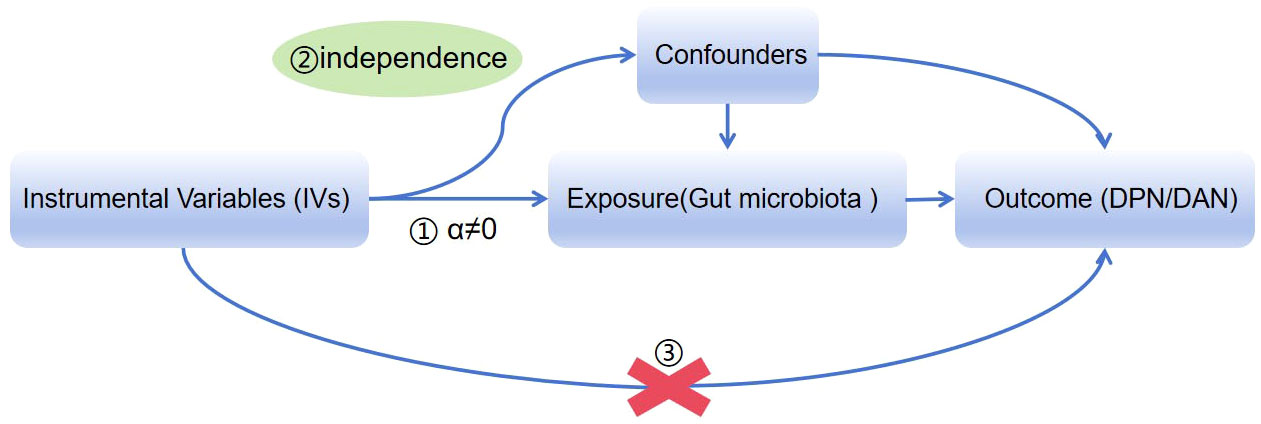
Three key assumptions for a valid Mendelian randomization study.

We obtained summary data from genome-wide association studies (GWASs) on gut microbiota and DPN/DAN. To ensure reliable results, our MR analysis adhered to three assumptions: (1) the instrumental variables (IVs) used must be closely related to the taxonomic groups of microbiota; (2) the IVs included and confounding factors (which affect the taxonomic groups of microbiota and diabetic neurodegeneration) must be independent of each other; (3) no horizontal pleiotropy: IVs only affect diabetic neurodegeneration through the taxonomic groups of microbiota (**Figure 1**) (11). Our study results were reported in accordance with the MR-STROBE guidelines.

### 2.2 Ethical statement

The summary-level data used in this study are publicly available and de-identified. The GWASs included have been approved by their respective institutions.

### 2.3 Exposure sources of gut microbiota data

Kurilshikov et al. conducted a large-scale, multi-ethnic, whole-genome meta-analysis to investigate the association between common human genetic variations and gut microbiota composition. This analysis was based on data from the MiBioGen consortium (12). The MiBioGen consortium comprised 18,340 individuals of European ethnicity from 11 cohorts across 25 countries. The GWAS study analyzed trans-ethnic taxonomic group variation and generated 110,211 variant sites from 122 taxa (from phylum to genus level). IVs for gut microbiota at the genus level were extracted from this large-scale GWAS. Additional details about the gut microbiota data are available in the original publication.

To ensure the robustness of the data and the accuracy of the results, SNPs underwent quality control to obtain eligible IVs: (1) Due to the small number of IVs meeting this criterion, a relatively comprehensive threshold (P<1×10^-5^) was used to obtain more comprehensive results (13). (2) To meet the MR assumption, linkage disequilibrium (LD) analysis was performed (R2<0.001, clustering distance=10,000kb) based on the 1000 Genomes Project of Europeans, and SNPs that did not meet the requirements were removed. (3) Palindromic SNPs were eliminated to mitigate the potential influence of allele confounding on the causal relationship between gut microbiota and diabetic neuropathy. The strength of the selected SNPs was assessed using the following formula to calculate the F-statistic for each bacterial taxonomic unit: F = R²(N-K-1)/K(1-R²), with R² representing the proportion of exposure variance explained by IVs, n denoting the sample size, and k indicating the number of IVs. An F-statistic >10 indicates that there is no presence of noticeable weak instrument bias (11).

### 2.4 Data sources for DPN and DAN

The GWAS summary statistics for DPN and DAN were obtained from the FinnGen research project (https://r9finngen.fi/). The DPN dataset consisted of 1,048 cases and 374,434 controls, while the DAN dataset included 111 cases and 374,434 controls, both sourced from the FinnGen consortium. DPN and DAN were defined based on electronic medical records and International Classification of Diseases (ICD) codes.

### 2.5 Statistical analysis

**Figure 2:**
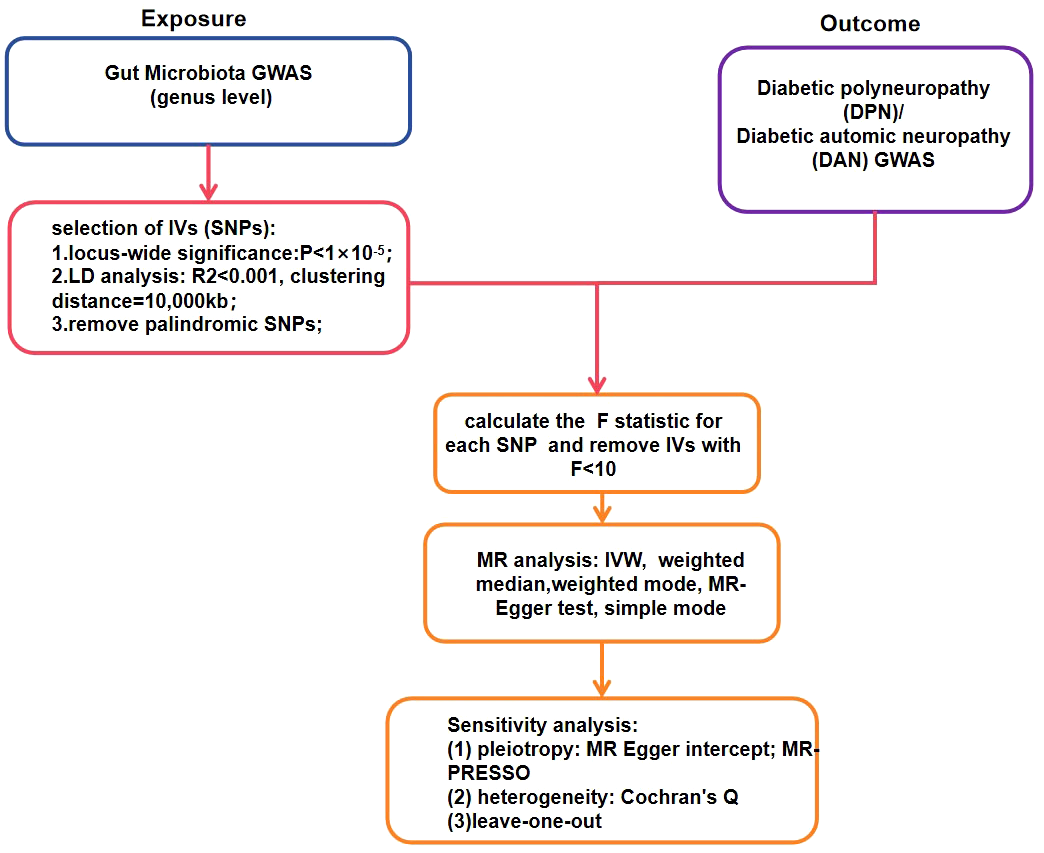
The flowchart of the Mendelian randomization study revealing the causal relationship between gut microbiota and DPN/DAN.

All statistical analyses were conducted using R software (version 4.1.1). We employed the “TwoSampleMR” R package for Mendelian randomization (MR) analysis to evaluate the causal relationships between gut microbiota and diabetic neuropathy. P-value< 0.05 was considered indicative of statistically significant evidence for potential causal effects (14). The study flowchart is presented in **Figure 2**.

#### 2.5.1 MR analysis

The Wald ratio method was used to test the influence of individual instrumental variables (IVs) on causal estimates. In the absence of horizontal pleiotropy, the inverse variance-weighted (IVW) test was adopted as the primary approach to compute unbiased estimates of causal effects. Effect sizes were reported as odds ratios (ORs) with corresponding 95% confidence intervals (CIs). Additional methods employed in the MR analysis included weighted median, weighted mode, MR-Egger test, and simple mode.

#### 2.5.2 Sensitivity analysis

The presence of heterogeneity was tested using Cochrane’s Q test. An IV with a P-value less than 0.05 was considered to exhibit significant heterogeneity. The MR-Egger intercept assessed the potential presence of pleiotropy in the IV. If P>0.05, then the absence of horizontal pleiotropy was determined. To further analyze pleiotropy and remove potential outliers, MR-Pleiotropy RESidual Sum and Outlier (MR-PRESSO) testing (R package “MR-PRESSO”) was utilized (15). Additionally, leave-one-out methods were employed to further validate the robustness of the data (16). Because there was an insufficient number of SNPs that met the MR research hypothesis, a reverse MR analysis was not conducted. To emphasize our contributions, we clearly identified the innovative aspects of our model and method in the abstract.

## 3 Results

### 3.1 Selection of IVs

**Figure 3:**
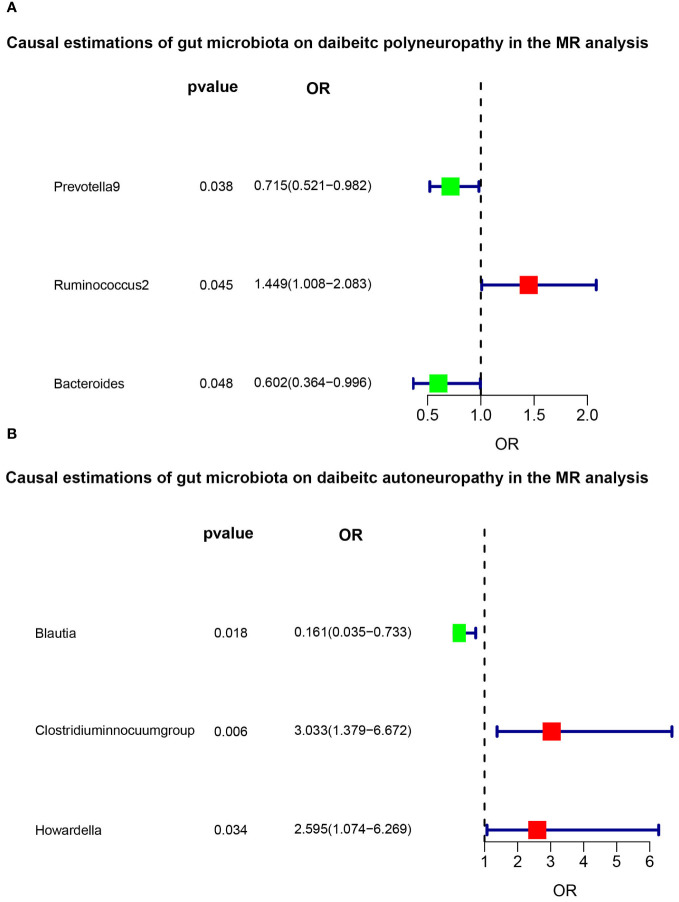
Significant results of MR analysis. The the green and red squares indicates decreased risk (OR<1) and increased risk (OR>1), respectively. **(A)** The main IVW method indicated that *Prevotella 9* had a protective effect on DPN (OR=0.715, 95%CI: 0.521-0.982, P=0.038), and *Bacteroides* also showed a protective effect (OR=0.602, 95%CI: 0.364-0.996, P=0.048). However, *Ruminococcus 2* had a promotive effect on DPN (OR=1.449, 95%CI: 1.008-2.083, P=0.045). **(B)** *Blautia* (OR=0.161, 95%CI=0.035-0.733, P=0.018), *Clostridium innocuum group* (OR=3.033, 95%CI=1.379-6.672, P=0.006), and *Howardella* (OR=2.595, 95%CI=1.074-6.269, P=0.034) were causally associated with DAN in the IVW analysis.

The F-statistic was computed for each single nucleotide polymorphism (SNP), all of which exceeded the threshold value of 10, indicating the strong instrumental validity. Based on the criteria for screening IVs, a total of 1532 SNPs were selected as IVs for 119 bacterial genera. **Tables 1**, **2** present the causal effects of gut microbiota on diabetic peripheral neuropathy (DPN) and diabetic autonomic neuropathy (DAN), respectively. We identified 119 bacterial genera, especially *Clostridium sensu stricto1*, *Prevotella 9*, *Ruminococcus 2*, *Ruminococcaceae UCG005*, *Bacteroides*, *Enterorhabdus*, and *Lachnoclostridium*, that were associated with DPN in at least one Mendelian randomization (MR) method. The inverse variance-weighted (IVW) estimate revealed a protective effect of *Prevotella 9* against DPN (OR=0.715, 95%CI: 0.521-0.982, P=0.038), whereas *Bacteroides* also showed a protective effect on DPN (OR=0.602, 95%CI: 0.364-0.996, P=0.048) and *Ruminococcus 2* had a protective effect on DPN (OR=1.449, 95%CI: 1.008-2.083, P=0.045). Additionally, no apparent directional horizontal pleiotropy was observed based on the MR-Egger regression intercept analysis shown in **Table 2**. Furthermore, IVW analysis revealed causal associations between *Blautia* (OR=0.161, 95%CI=0.035-0.733, P=0.018), *Clostridium innocuum group* (OR=3.033, 95%CI=1.379-6.672, P=0.006), and *Howardella* (OR=2.595, 95%CI=1.074-6.269, P=0.034) and DAN, without any evidence of heterogeneity or pleiotropy (**Figure 3**).

**Table 1:** Significant Mendelian randomization estimates of the association between bacterial genera and DPN(P<0.05).

Microbiota genus	NSNPs	MR method	OR	95% CI	P Value	pleiotropy			heterogeneity	
						Egger intercept	SE	P value	Q	P value
Prevotella 9	17	MR Egger	0.804	0.323-1.999	0.646	-0.012	0.045	0.790	18.610	0.531
		Weighted median	0.825	0.547-1.246	0.362					
		IVW	0.715	0.520-0.982	0.038				8.182	0.611
		Simple mode	0.835	0.411-1.694	0.625					
		Weighted mode	0.865	0.476-1.570	0.641					
Ruminococcus 2	15	MR Egger	1.786	0.744-4.285	0.216	-0.018	0.035	0.615	10.944	0.615
		Weighted median	1.384	0.807-2.3744	0.236					
		IVW	1.449	1.008-2.082	0.045				11.209	0.669
		Simple mode	1.469	0.620-3.483	0.396					
		Weighted mode	1.320	0.667-2.611	0.437					
Bacteroides	11	MR Egger	1.034	0.068	0.981	-0.035	0.088	0.699	8.023	0.531
		Weighted median	0.673	0.340-1.332	0.256					
		IVW	0.602	0.363-0.996	0.048				8.182	0.611
		Simple mode	0.771	0.257-2.311	0.653					
		Weighted mode	0.688	0.245-1.929	0.494					

**Table 2:** Significant Mendelian randomization estimates of the association between bacterial genera and DAN(P<0.05).

Microbiota genus	NSNPs	MR method	OR	95% CI	P Value	pleiotropy			heterogeneity	
						Egger intercept	SE	P value	Q	P value
Blautia	12	MR Egger	0.115	0.003-5.053	0.289	0.026	0.137	0.854	9.930	0.447
		Weighted median	0.102	0.015-0.712	0.021					
		IVW	0.161	0.035-0.733	0.018				9.965	0.534
		Simple mode	0.083	0.004-1.784	0.140					
		Weighted mode	0.072	0.003-1.843	0.140					
Clostridium innocuum group	15	MR Egger	27.895	0.439-1771.991	0.155	-0.296	0.277	0.317	6.407	0.602
		Weighted median	1.532	0.536-4.377	0.426					
		IVW	3.033	1.379-6.672	0.006				7.546	0.580
		Simple mode	1.188	0.184-7.689	0.860					
		Weighted mode	1.218	0.200-7.412	0.835					
Howardella	11	MR Egger	0.089	0.004-1.937	0.162	0.532	0.241	0.058	8.187	0.415
		Weighted median	1.776	0.627-5.031	0.280					
		IVW	2.595	1.074-6.269	0.034			13.189	0.154
		Simple mode	3.460	0.415-28.840	0.281					
		Weighted mode	0.841	0.189-3.751	0.826					

### 3.2 sensitivity analysis

**Figure 4:**
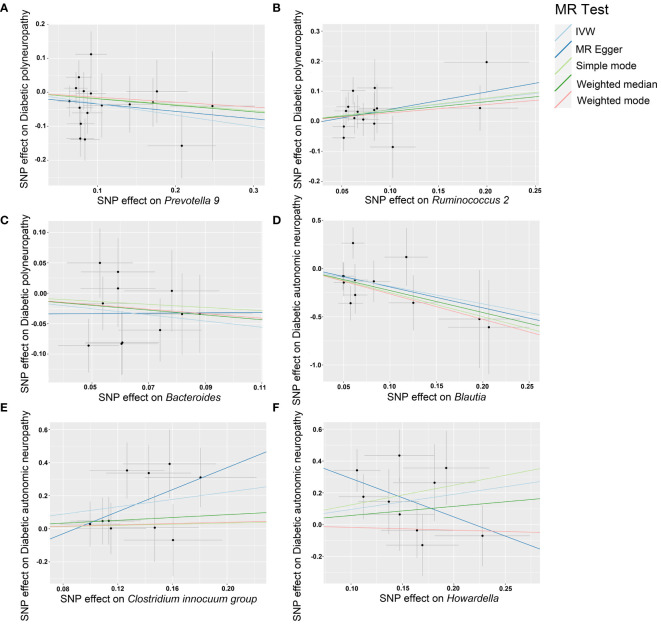
Scatter plots of each genus associated with the risk of DPN and DAN, respectively. **(A)** *Prevotella 9*. **(B)** *Ruminococcus 2.* **(C)** *Bacteroides.* **(D)** *Blautia*. **(E)** *Clostridium innocuum group*. **(F)** *Howardella*.

**Figure 5:**
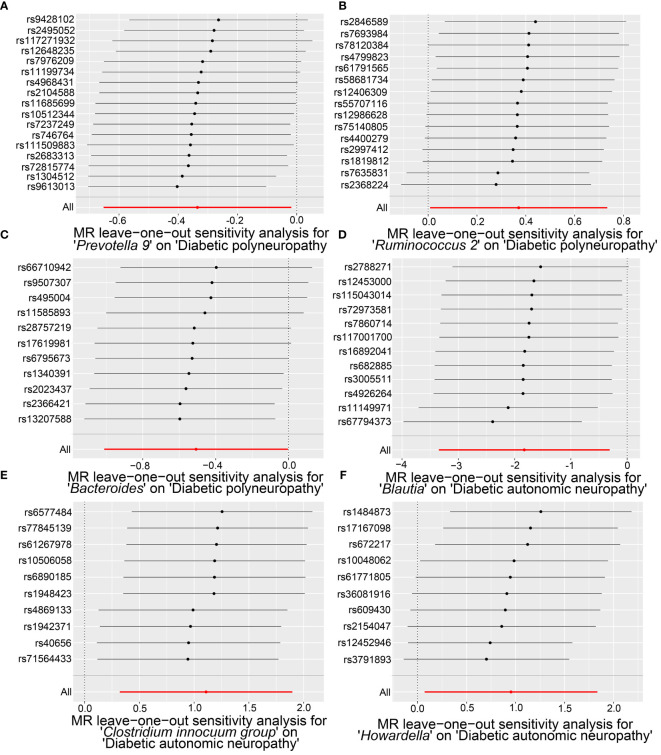
The “leave-one-out” analysis results of significant microbial taxa on the causal effects of DPN and DAN, revealing minimal changes in the overall error bars when each SNP is removed. Specifically, all error bars either remain to the right of 0 or to the left of 0, indicating the absence of outliers. **(A)** The causal effect of *Prevotella 9* on DPN. **(B)** The causal effect of *Ruminococcus 2* on DPN. **(C)** The causal effect of *Bacteroides* on DPN. **(D)** The causal effect of *Blautia* on DAN. **(E)** The causal relationship of the *Clostridium innocuum group* on DAN. **(F)** The causal effect of *Howardella* on DAN.

Our sensitivity analysis revealed no heterogeneity in the IV for any of the six bacterial genera (**Tables 1**, **2**), indicating that the genetic traits associated with these genera may be caused by changes in multiple genetic materials. The MR-Egger regression intercept indicated no evidence of horizontal pleiotropy for any of the six bacterial genera (**Tables 1**, **2**) with a P-value greater than 0.05. The scatter plots (**Figure 4**) revealed that *Prevotella 9* and *Bacteroides* may have a protective effect on DPN, while *Ruminococcus 2* may have a promotive effect. Additionally, *Blautia* may have a protective effect on DAN, while *Clostridium innocuum group* and *Howardella* may have a promotive effect. The scatter plot illustrates the following MR analysis methods in order: the inverse-variance weighted (IVW) method, MR-Egger, weighted median, weighted mode, and simple mode. In **Figure 4**, the lines moving up from left to right represent the promotive indicator of the relationship between the genus and the disease, while the lines moving down from left to right represent the protective indicator. Our “leave-one-out” analysis revealed no potential outliers in the IV for any of the six bacterial genera, suggesting that all identified causal relationships were robust and not affected by individual IVs (**Figure 5**).

## 4 Discussion

We conducted a two-sample Mendelian randomization (MR) analysis using summary statistics data from the largest-scale GWAS meta-analysis of gut microbiota conducted by the MiBioGen consortium. We also used summary statistics data from the FinnGen consortium R8 release data on diabetic polyneuropathy (DPN) and diabetic autonomic neuropathy (DAN). Our aim was to establish the genetic causal relationship between gut microbiota and diabetic neuropathy. Our findings indicate that higher genetic abundance of two gut microbial taxa (genus *Prevotella 9* and genus *Bacteroides*) is associated with a decreased risk of DPN, whereas higher genetic abundance of genus *Ruminococcus 2* is linked to an increased risk of DPN. Furthermore, we identified genus *Blautia* and genus *Clostridium innocuum group*, as potential risk factors for DAN development. Importantly, our study is the first to employ the MR concept in examining the causal relationship between gut microbiota and diabetic neuropathy. This enables the implementation of secondary prevention strategies for individuals at risk of DPN or DAN. Targeted screening of gut microbiota can be conducted for populations at risk of DPN or DAN, such as patients with type 2 diabetes and metabolic syndrome. This screening enables early disease detection and facilitates the establishment of treatment mechanisms. We also encourage individuals to maintain a healthy diet, regularly use suitable probiotic supplements to preserve gut microbiota balance, and ensure normal short-chain fatty acid metabolism, effectively preventing diabetic neuropathy and its complications.

Many studies have demonstrated that changes in gut microbiota composition and its metabolites are associated with obesity and metabolic disorders and represent potential therapeutic targets for these diseases (17, 18). Genus *Prevotella*, consists of anaerobic bacteria that produce acetate, succinate, isovalerate, and lactate as their primary fermentation products. Previous research has indicated that *Prevotella* bacteria are also involved in human glucose metabolism. For instance, rat glucose tolerance improved after gut perfusion with *Prevotella* bacteria (19). An increase in *Prevotella* abundance modulates the gut-pancreas axis and blood sugar levels via its metabolites, short-chain fatty acids, and by promoting glucagon-like peptide-1 (GLP-1) release in intestinal L cells, which is consistent with our findings (20).

Additionally, oligofructose has been shown to alleviate symptoms of type 2 diabetes by modulating the abundance of genus *Bacteroides*, which impacts the pathways of “linoleic acid metabolism,” “serotonin synapse,” and “tryptophan metabolism” (21). Genus *Bacteroides*, is known for producing bile salt hydrolase, which aids in bile acid metabolism in the gut and can transform bile acids to increase secondary bile acid content, including taurochenodeoxycholic acid. Research has also demonstrated that taurochenodeoxycholic acid enhances the cell viability and migration ability of high glucose-induced Schwann cells, potentially exerting a protective effect against diabetic neuropathy through this pathway (22–26).

Previous research has demonstrated that an increase in the abundance of genus *Blautia* is linked to improved glucose and lipid homeostasis (27). Furthermore, *Blautia* is a common acetate producer in the gut, activating G-protein coupled receptors (GPR41 and GPR43) to inhibit insulin signaling and fat accumulation in adipocytes, promoting the metabolism of unbound lipids and glucose in other tissues, and thereby alleviating obesity-related diseases (28). *Blautia wexlerae*’s beneficial effects are due to its unique amino acid metabolism, which produces S-adenosylmethionine, acetylcholine, L-ornithine, and accumulates branched starch, as well as succinate, lactate, and acetate salts, thereby altering gut bacteria composition. These findings reveal novel regulatory pathways in host-microbe metabolism, which could offer new approaches to metabolic disorder prevention and treatment (29).

The study demonstrates that Huanglian treatment can modify microbial bile acid metabolism, which improves impaired glucose tolerance and lipid accumulation in T2DM mice. These improvements may be attributed, in part, to modulation of the gut microbiota and bile acid pool structure, suggesting that Huanglian has beneficial effects on T2DM mediated by the gut microbiota (30). Additionally, this study found a positive correlation between neurogenic intestinal dysfunction and genus *Clostridium innocuum*, supporting previous research (31).

Previous research has overlooked *Howardella*, but reports suggest that an increased abundance of *Howardella* is linked to prediabetes and diabetes (32). Moreover, oligomannose reduces or reverses the increased abundance of *Howardella* observed in healthy or prediabetic subjects, potentially preventing changes in gut microbiota during diabetes development (33).

There is also a growing amount of researches indicating a close connection between the gut microbiota and the nervous system. The gut microbiota may play a pivotal role as predisposing factors for neurological diseases, including Alzheimer’s disease, autism spectrum disorders, multiple sclerosis, Parkinson’s disease, and stroke (34). Additionally, bacteria in the gastrointestinal tract can activate neural pathways and central nervous system signaling systems, affecting stress-related behaviors such as anxiety and depression (35). Furthermore, evidence also associates dysbiosis of the gut microbiota with hypertension through the autonomic nervous system (36). Studies indicate that gut bacteria play a significant role in chemotherapy-induced peripheral neuropathy (37). Researches have demonstrated that Paeoniflorin and Danggui Sini decoction can alleviate oxaliplatin-induced peripheral neuropathy by modulating the gut microbiota (38, 39). And administration of a young gut microbiota to elderly mice has been found to improve nerve repair and enhance functional recovery following peripheral nerve damage (40). There is also research that has shown partial restoration of the gut microbiota can alleviate neuropathic pain caused by nerve damage, chemotherapy, and diabetic neuropathy (41).

This study has several advantages. Firstly, previous observational studies have faced challenges in controlling confounding factors such as age, gender, and lifestyle, which has led to lower reliability and accuracy of experimental results. Secondly, the utilization of the latest large-scale GWAS enables the acquisition of genetic data and analysis from a substantial sample population, thereby enhancing the reliability of research results in comparison to small-scale case-control studies. Moreover, MR analysis mitigates confounding and offers a novel approach to investigating the “gut-neuro axis” mechanism.

It is essential to acknowledge the limitations of this study. Firstly,the inclusion of only participants of European ancestry in the GWAS raises concerns about the generalizability of the study’s results to other racial/ethnic groups. Secondly, the sample size of DAN is relatively small, potentially leading to imprecise estimates and limited statistical power to detect causal effects. Thirdly, there may be unmeasured confounders not accounted for in the analysis, leading to the existence of residual confounding and potentially causing flaws in the validity of our findings. Henceforth, the results should be interpreted carefully. While Mendelian randomization can provide evidence for causal relationships, it is important to seek additional sources of evidence to support the causal claims. Furthermore, investigating the intermediate role of blood proteins and metabolites in the pathway from environmental exposure to diabetes is crucial to provide evidence for treatment and intervention.

## 5 Conclusion

Our study indicated that genus *Prevotella 9* and genus *Bacteroides* had a protective effect on DPN, while genus *Ruminococcus 2* had a promoting effect. And genus *Blautia*, genus *Clostridium innocuum group*, and genus *Howardella* were causally associated with DAN. These findings identified a causal relationship between gut microbiota and the increased or decreased risk of diabetic neuropathy, which may underscore the importance of adopting a comprehensive approach that combines gut microbiota modulation with other therapeutic interventions in the management of diabetic neuropathy.

## Data availability statement

Publicly available datasets were analyzed in this study. This data can be found here: https://storage.googleapis.com/finngen-public-data-r9/summary_stats/finngen_R9_DM_AUTONOMIC.gz https://storage.googleapis.com/finngen-public-data-r9/summary_stats/finngen_R9_DM_POLYNEURO.gz.

## Ethics statement

Ethical approval was not required for the study involving humans in accordance with the local legislation and institutional requirements. Written informed consent to participate in this study was not required from the participants or the participants’ legal guardians/next of kin in accordance with the national legislation and the institutional requirements.

## Author contributions

MX: Data curation, Investigation, Methodology, Software, Validation, Visualization, Writing – original draft, Writing – review & editing. JH: Investigation, Resources, Software, Writing – original draft, Writing – review & editing. YQ: Data curation, Formal analysis, Investigation, Software, Writing – original draft, Writing – review & editing. BW: Investigation, Validation, Writing – original draft, Writing – review & editing. RL: Visualization, Writing – original draft, Writing – review & editing. XY: Investigation, Writing – original draft, Writing – review & editing. YZ: Conceptualization, Methodology, Supervision, Validation, Writing – original draft, Writing – review & editing. YL: Conceptualization, Data curation, Investigation, Methodology, Project administration, Software, Supervision, Validation, Writing – original draft, Writing – review & editing.

## References

[1] Rojas DR Tegeder I Kuner R Agarwal N. Hypoxia-inducible factor 1α protects peripheral sensory neurons from diabetic peripheral neuropathy by suppressing accumulation of reactive oxygen species. J Mol Med (Berl). (2018) 96:1395–405. doi: 10.1007/s00109-018-1707-9 30361814

[2] Rauskolb S Dombert B Sendtner M. Insulin-like growth factor 1 in diabetic neuropathy and amyotrophic lateral sclerosis. Neurobiol Dis. (2017) 97:103–13. doi: 10.1016/j.nbd.2016.04.007 27142684

[3] Yang X Yao W Liu H Gao Y Liu R Xu L. Tangluoning, a traditional Chinese medicine, attenuates *in vivo* and *in vitro* diabetic peripheral neuropathy through modulation of PERK/Nrf2 pathway. Sci Rep. (2017) 7:1014. doi: 10.1038/s41598-017-00936-9 28432299 PMC5430716

[4] Javed S Alam U Malik RA. Burning through the pain: treatments for diabetic neuropathy. Diabetes Obes Metab. (2015) 17:1115–25. doi: 10.1111/dom.12535 26179288

[5] Zhao L Zhang Q Ma W Tian F Shen H. Zhou M. A combination quercetin resveratrol reduces Obes high-fat diet-fed rats by modulation gut microbiota. Food Funct. (2017) 8:4644–56. doi: 10.1039/c7fo01383c 29152632

[6] Anlu W Dongcheng C He Z Qiuyi L Yan Z Yu Q. Using herbal medicine to target the "microbiota-metabolism-immunity" axis as possible therapy for cardiovascular disease. Pharmacol Res. (2019) 142:205–22. doi: 10.1016/j.phrs.2019.02.018 30794922

[7] Yang G Wei J Liu P Zhang Q Tian Y Hou G. Role of the gut microbiota in type 2 diabetes and related diseases. Metabolism. (2021) 117:154712. doi: 10.1016/j.metabol.2021.154712 33497712

[8] Grasset E Puel A Charpentier J Collet X Christensen JE Tercé F. A specific gut microbiota dysbiosis of type 2 diabetic mice induces GLP-1 resistance through an enteric NO-dependent and gut-brain axis mechanism. Cell Metab. (2017) 25:1075–1090.e5. doi: 10.1016/j.cmet.2017.04.013 28467926

[9] Yang J Yang X Wu G Huang F Shi X Wei W. Gut microbiota modulate distal symmetric polyneuropathy in patients with diabetes. Cell Metab. (2023) 35:1548–1562.e7. doi: 10.1016/j.cmet.2023.06.010 37451270

[10] Lawlor DA. Commentary: Two-sample Mendelian randomization: opportunities and challenges. Int J Epidemiol. (2016) 45:908–15. doi: 10.1093/ije/dyw127 PMC5005949 27427429

[11] Lawlor DA Harbord RM Sterne JA Timpson N Davey Smith G. Mendelian randomization: using genes as instruments for making causal inferences in epidemiology. Stat Med. (2008) 27:1133–63. doi: 10.1002/sim.3034 17886233

[12] Kurilshikov A Medina-Gomez C Bacigalupe R Radjabzadeh D Wang J Demirkan A. Large-scale association analyses identify host factors influencing human gut microbiome composition. Nat Genet. (2021) 53:156–65. doi: 10.1038/s41588-020-00763-1 PMC8515199 33462485

[13] Bowden J Holmes MV. Meta-analysis and Mendelian randomization: A review. Res Synth Methods. (2019) 10:486–96. doi: 10.1002/jrsm.1346 PMC6973275 30861319

[14] Hemani G Zheng J Elsworth B Wade KH Haberland V Baird D. The MR-Base platform supports systematic causal inference across the human phenome. Elife. (2018) 7:e34408. doi: 10.7554/eLife.34408 29846171 PMC5976434

[15] Verbanck M Chen CY Neale B Do R. Detection of widespread horizontal pleiotropy in causal relationships inferred from Mendelian randomization between complex traits and diseases. Nat Genet. (2018) 50:693–8. doi: 10.1038/s41588-018-0099-7 PMC6083837 29686387

[16] Hemani G Tilling K Davey Smith G. Orienting the causal relationship between imprecisely measured traits using GWAS summary data. PloS Genet. (2017) 13:e1007081. doi: 10.1371/journal.pgen.1007081 29149188 PMC5711033

[17] Belkaid Y Hand TW. Role of the microbiota in immunity and inflammation. Cell. (2014) 157:121–41. doi: 10.1016/j.cell.2014.03.011 PMC4056765 24679531

[18] Belkaid Y Harrison OJ. Homeostatic immunity and the microbiota. Immunity. (2017) 46:562–76. doi: 10.1016/j.immuni.2017.04.008 PMC5604871 28423337

[19] Péan N Le Lay A Brial F Wasserscheid J Rouch C Vincent M. Dominant gut Prevotella copri in gastrectomised non-obese diabetic Goto-Kakizaki rats improves glucose homeostasis through enhanced FXR signalling. Diabetologia. (2020) 63:1223–35. doi: 10.1007/s00125-020-05122-7 PMC7228998 32173762

[20] Le Bourgot C Ferret-Bernard S Apper E Taminiau B Cahu A Le Normand L. Perinatal short-chain fructooligosaccharides program intestinal microbiota and improve enteroinsular axis function and inflammatory status in high-fat diet-fed adult pigs. FASEB J. (2019) 33:301–13. doi: 10.1096/fj.201800108R 29975568

[21] Li P Tong T Wu Y Zhou X Zhang M Liu J. The Synergism of Human Lactobacillaceae and Inulin Decrease Hyperglycemia via Regulating the Composition of Gut Microbiota and Metabolic Profiles in db/db Mice. J Microbiol Biotechnol. (2023) 33:1–14. doi: 10.4014/jmb.2304.04039 37734909 PMC10772568

[22] Kawamoto K Horibe I Uchida K. Purification and characterization of a new hydrolase for conjugated bile acids, chenodeoxycholyltaurine hydrolase, from Bacteroides vulgatus. J Biochem. (1989) 106:1049–53. doi: 10.1093/oxfordjournals.jbchem.a122962 2628421

[23] Coleman JP Hudson LL. Cloning and characterization of a conjugated bile acid hydrolase gene from Clostridium perfringens. Appl Environ Microbiol. (1995) 61:2514–20. doi: 10.1128/aem.61.7.2514-2520.1995 PMC167523 7618863

[24] Wolf PG Devendran S Doden HL Ly LK Moore T Takei H. Berberine alters gut microbial function through modulation of bile acids. BMC Microbiol. (2021) 21:24. doi: 10.1186/s12866-020-02020-1 33430766 PMC7798349

[25] Wang Y Ye X Ding D Lu Y. Characteristics of the intestinal flora in patients with peripheral neuropathy associated with type 2 diabetes. J Int Med Res. (2020) 48:300060520936806. doi: 10.1177/0300060520936806 32938282 PMC7503028

[26] Wang Q Li W Zhang X Chung SL Dai J Jin Z. Tauroursodeoxycholic acid protects Schwann cells from high glucose-induced cytotoxicity by targeting NLRP3 to regulate cell migration and pyroptosis. Biotechnol Appl Biochem. (2024) 71(1):28–37. doi: 10.1002/bab.2518 37749820

[27] Tong X Xu J Lian F Yu X Zhao Y Xu L. Structural alteration of gut microbiota during the amelioration of human type 2 diabetes with hyperlipidemia by metformin and a traditional chinese herbal formula: a multicenter, randomized, open label clinical trial. mBio. (2018) 9:e02392–17. doi: 10.1128/mBio.02392-17 PMC5964358 29789365

[28] Kimura I Ozawa K Inoue D Imamura T Kimura K Maeda T. The gut microbiota suppresses insulin-mediated fat accumulation via the short-chain fatty acid receptor GPR43. Nat Commun. (2013) 4:1829. doi: 10.1038/ncomms2852 23652017 PMC3674247

[29] Hosomi K Saito M Park J Murakami H Shibata N Ando M. Oral administration of Blautia wexlerae ameliorates obesity and type 2 diabetes via metabolic remodeling of the gut microbiota. Nat Commun. (2022) 13:4477. doi: 10.1038/s41467-022-32015-7 35982037 PMC9388534

[30] Li D Feng G Li Y Pan H Luo P Liu B. Benefits of Huang Lian mediated by gut microbiota on HFD/STZ-induced type 2 diabetes mellitus in mice. Front Endocrinol (Lausanne). (2023) 14:1120221. doi: 10.3389/fendo.2023.1120221 36742405 PMC9889990

[31] Rendeli C Paradiso VF Bucci V Cretì G D'Aleo C Lisi G. Gut microbiota and pediatric patients with spina bifida and neurogenic bowel dysfunction. Childs Nerv Syst. (2023) 39:633–45. doi: 10.1007/s00381-022-05688-0 36180597

[32] Yang J Summanen PH Henning SM Hsu M Lam H Huang J. Xylooligosaccharide supplementation alters gut bacteria in both healthy and prediabetic adults: a pilot study. Front Physiol. (2015) 6:216. doi: 10.3389/fphys.2015.00216 26300782 PMC4528259

[33] Liu CZ Chen W Wang MX Wang Y Chen LQ Zhao F. Dendrobium officinale Kimura et Migo and American ginseng mixture: A Chinese herbal formulation for gut microbiota modulation. Chin J Nat Med. (2020) 18:446–59. doi: 10.1016/S1875-5364(20)30052-2 32503736

[34] Cryan JF O'Riordan KJ Sandhu K Peterson V Dinan TG. The gut microbiome in neurological disorders. Lancet Neurol. (2020) 19:179–94. doi: 10.1016/S1474-4422(19)30356-4 31753762

[35] Foster JA McVey NKA. Gut-brain axis: how the microbiome influences anxiety and depression. Trends Neurosci. (2013) 36:305–12. doi: 10.1016/j.tins.2013.01.005 23384445

[36] Zubcevic J Richards EM Yang T Kim S Sumners C Pepine CJ. Impaired autonomic nervous system-microbiome circuit in hypertension. Circ Res. (2019) 125:104–16. doi: 10.1161/CIRCRESAHA.119.313965 PMC6588177 31219753

[37] Ramakrishna C Corleto J Ruegger PM Logan GD Peacock BB Mendonca S. Dominant role of the gut microbiota in chemotherapy induced neuropathic pain. Sci Rep. (2019) 9:20324. doi: 10.1038/s41598-019-56832-x 31889131 PMC6937259

[38] Xu J Lu L Jiang S Qin Z Huang J Huang M. Paeoniflorin ameliorates oxaliplatin-induced peripheral neuropathy via inhibiting neuroinflammation through influence on gut microbiota. Eur J Pharmacol. (2024) 971:176516. doi: 10.1016/j.ejphar.2024.176516 38513881

[39] Chen C Xu JL Gu ZC Zhou SS Wei GL Gu JL. Danggui Sini decoction alleviates oxaliplatin-induced peripheral neuropathy by regulating gut microbiota and potentially relieving neuroinflammation related metabolic disorder. Chin Med. (2024) 19:58. doi: 10.1186/s13020-024-00929-7 38584284 PMC10999090

[40] Svačina MKR Gao T Sprenger-Svačina A Lin J Ganesh BP Lee J等. Rejuvenating fecal microbiota transplant enhances peripheral nerve repair in aged mice by modulating endoneurial inflammation. Exp Neurol. (2024) 376:114774. doi: 10.1016/j.expneurol.2024.114774 38599367

[41] Ma P Mo R Liao H Qiu C Wu G Yang C. Gut microbiota depletion by antibiotics ameliorates somatic neuropathic pain induced by nerve injury, chemotherapy, and diabetes in mice. J Neuroinflamm. (2022) 19:169. doi: 10.1186/s12974-022-02523-w PMC9237999 35764988

